# Common signatures of selection reveal target loci for breeding across soybean populations

**DOI:** 10.1002/tpg2.20426

**Published:** 2024-01-23

**Authors:** João Paulo Gomes Viana, Arián Avalos, Zhihai Zhang, Randall Nelson, Matthew E. Hudson

**Affiliations:** ^1^ National Center for Supercomputing Applications University of Illinois at Urbana‐Champaign Urbana Illinois USA; ^2^ U. S. Department of Agriculture, Honeybee Breeding, Genetics, and Physiology Research Baton Rouge Louisiana USA; ^3^ DOE Center for Advanced Bioenergy and Bioproducts Innovation (CABBI) University of Illinois at Urbana‐Champaign Urbana Illinois USA; ^4^ USDA‐ARS, Department of Crop Sciences, University of Illinois at Urbana‐Champaign Urbana Illinois USA; ^5^ Department of Crop Sciences University of Illinois at Urbana‐Champaign 1102 S Goodwin Ave Urbana Illinois 61801 USA

## Abstract

Understanding the underlying genetic bases of yield‐related selection and distinguishing these changes from genetic drift are critical for both improved understanding and future success of plant breeding. Soybean [*Glycine max* (L.) Merr.] is a key species for world food security, yet knowledge of the mechanism of selective breeding in soybean, such as the century‐long program of artificial selection in U.S. soybean germplasm, is currently limited to certain genes and loci. Here, we identify genome‐wide signatures of selection in separate populations of soybean subjected to artificial selection for increased yield by multiple breeding programs in the United States. We compared the alternative soybean breeding population (AGP) created by USDA‐ARS to the conventional public soybean lines (CGP) developed at three different stages of breeding (ancestral, intermediate, and elite) to identify shared signatures of selection and differentiate these from drift. The results showed a strong selection for specific haplotypes identified by single site frequency and haplotype homozygosity methods. A set of common selection signatures was identified in both AGP and CGP that supports the hypothesis that separate breeding programs within similar environments coalesce on the fixation of the same key haplotypes. Signatures unique to each breeding program were observed. These results raise the possibility that selection analysis can allow the identification of favorable alleles to enhance directed breeding approaches.

## Core Ideas

- Specific loci are under selection for key traits during soybean breeding.
- These loci can be detected and mapped by analysis of signatures of selection in the population.
- Signatures of drift versus selection can be differentiated by the use of parallel, separate populations that are under selection for the same traits in the same environment.
- Identification of desirable traits from different populations can add to the overall diversity of the elite soybean gene pool.

**Abbreviations**
AGP: alternative gene pool
CGP: conventional gene pool
DAPC: discriminant analysis of principal components
EHH: extended haplotype homozygosity
GWAS: genome‐wide association study
LD: linkage disequilibrium
LDA: linear discriminant analysis
MG: maturity group
QTL: quantitative trait locus
SFS: site frequency spectrum
SNP: single nucleotide polymorphism

## 1 INTRODUCTION

Soybean is a crop that has become one of the most important oil and protein sources for human and animal food. Due to its spectrum of industry and food applications, soybean is also considered an important commercial commodity. As the population expands, the demand for grains and derivatives increases worldwide. In 2023, Brazil was the largest soybean producer, with an annual production of 163 million metric tons (mt), followed by the United States with an annual production of 111.7 mt and Argentina with an annual production of 48 mt (FAS/USDA, 2023). Soybean breeding programs have been driven by growing demand to develop increasingly productive and competitive lines. The evolutionary and selective breeding processes that led from the domestication of wild soybean [*Glycine max* subsp. *soja* (Siebold & Zucc.) H.Ohashi] in East Asia to the development of modern superior soybean lines involved a series of complex genomic changes in these populations (Jeong et al., 2019; Kim et al., 2012).

These genomic changes can be studied with the aid of molecular markers that allow access to the genetic diversity in populations at the molecular level (Guan et al., 2010; Zhang et al., 2013). In recent decades, advances in genotyping arrays and desoxyribonucleic acid (DNA) sequencing technologies have enabled the exploration of genetic diversity within large populations at greater precision, using relatively low‐cost markers based on single nucleotide polymorphisms (SNPs).

Deep sampling of multiple populations allows effective discrimination of two types of variations in allelic frequency: variation which occurs randomly in genomes due to demographic changes in the population, such as genetic drift, and variation which occurs due to specific phenomena, such as selection (Holderegger et al., 2006). In soybean breeding, positive selection for alleles of economic interest is expected to reduce genetic diversity in specific genomic regions, increasing the differentiation between populations, the linkage disequilibrium, and the homozygosity in these regions. Such selection usually leads to the fixation of the favorable allele and a selective sweep. However, studying genomic changes caused by selective breeding in crops is challenging because it is hard to differentiate between alleles under selection and those caused by drift in necessarily small breeding populations. The analysis of soybean germplasm in this way is particularly demanding due to the inbreeding nature of soybean and the low genetic diversity observed in soybean populations, even relative to other inbreeding crops (Viana et al., 2022). The frequency of a given allele that is not subject to selection will frequently increase or decrease purely by chance due to genetic drift (Kimura, 1968), and this is exacerbated in populations of small effective size, such as soybean breeding populations (Vaughn & Li, 2016; Xavier et al., 2018). The genetic drift in small breeding populations is expected to drive the fixation of non‐causal alleles, confounding analysis of the regions under selection.

The challenge in identifying signatures of selection in soybean germplasm is also related to the genetic basis of the target trait. In selective breeding for monogenic characters, such as many examples of resistance to pests or disease, the selection is detectable by the abrupt increase in allele frequencies in the regions that control the target trait. However, the detection of this process is more complicated in a scenario of selection for polygenic traits (Höllinger et al., 2019), such as yield‐related traits. In this last scenario, different combinations of relatively small‐effect favorable alleles can result in similar productivity levels, making it difficult to understand the genetic architecture of the target trait. In addition, breeders purposely maintain diversity within populations to maintain future genetic gain. Following this premise, we hypothesized that when considering elite soybean lines developed by different breeding programs, genomic regions responsible for increasing productivity are likely not entirely fixed in the populations of elite lines developed by these breeding programs but undergo a soft selective sweep. Therefore, we performed this study aimed to identify and compare the signatures of selection in a breeding program that incorporated exotic germplasm not otherwise used in cultivar development and a composite of public breeding programs in the northern United States. Both aim to obtain higher yielding soybean lines in the same region of the United States. We planned to compare them to differentiate the haplotypes proceeding to fixation as a result of drift, identify any haplotypes under common selection pressure in both gene pools, and identify any selected haplotypes that are unique to each gene pool. Common signatures of selection would be strong candidates for loci advantageous for overall yield, either by protecting the plants from biotic or abiotic stress or altering their physiology to produce higher seed yield. Those different signatures of selection between the gene pools indicate genetic variation that could be exploited to increase the yield of commercial soybean cultivars.

Therefore, the objective of this study was to identify haplotypes under positive selection in independent soybean breeding populations through different eras of U.S. soybean improvement.

## 2 MATERIALS AND METHODS

### 2.1 Plant material

Soybean lines from three different breeding stages (termed ancestral, intermediate, and elite, see Viana et al., 2022 for details) were sourced from the United States Department of Agriculture (USDA) Soybean Germplasm Collection and breeding collections of experimental lines developed by USDA‐ARS in Urbana, IL, in a program that forms a separate, alternative breeding population. The lines were stratified in two gene pools, according to the origin of the material (Table 1).

**TABLE 1:** Summary of gene pools, populations, and sample sizes.

Gene pool	Breeding stage	Population	Sample size
Alternative	Ancestral	Aa	43
	Intermediate	Ai	80
	Elite	Ae	68
Conventional	Ancestral	Ca	41
	Intermediate	Ci	61
	Elite	Ce	64
Total			357

Abbreviations: Aa, alternative gene pool—ancestral population; Ae, alternative gene pool—elite population; Ai, alternative gene pool—intermediate population; Ca, conventional gene pool—ancestral population; Ce, conventional gene pool—elite population; Ci, conventional gene pool—intermediate population.

*Source*: Viana et al. (2022).

The conventional gene pool (CGP) is composed of plant introductions, and commercial lines developed and released by several public breeding programs in the northern United States are composed of lines currently available from the USDA Soybean Germplasm Collection. The alternative gene pool (AGP) is formed by a different set of plant introductions corresponding to the ancestral lines and a set of high‐yielding soybean lines developed separately from the CGP over nearly four decades in the USDA breeding program at the University of Illinois at Urbana‐Champaign, IL (Table S1; Viana et al., 2022). The selected elite lines belong mainly to maturity groups (MG) III and MG IV. Some lines from the CGP were used as parents in the AGP.

**Core Ideas**

- Specific loci are under selection for key traits during soybean breeding.
- These loci can be detected and mapped by analysis of signatures of selection in the population.
- Signatures of drift versus selection can be differentiated by the use of parallel, separate populations that are under selection for the same traits in the same environment.
- Identification of desirable traits from different populations can add to the overall diversity of the elite soybean gene pool.

### 2.2 Genotyping and data processing

The DNA of AGP soybean lines from all breeding stages and CGP elite lines were extracted from leaf tissue as presented in the “Fosmid library construction” section in Cook et al., 2012, and the samples were genotyped using the SoySNP50K Infinium Chip (Song et al., 2013) at USDA‐ARS at Beltsville, MD. Data from ancestral and intermediate CGP lines, previously sequenced by Song et al. (2015), were retrieved from the SoyBase (Grant et al., 2010) Data Repository.

We mapped the SNPs based on the Williams 82 version 2 (Wm82.a2.v1) reference genome. Markers mapped to unplaced scaffolds were removed from the raw data set. To ensure data accuracy, we maintained a minimum call rate of 98% and treated heterozygous calls in the raw data set as missing data. Our filtering process utilized the R package *SNPRelate* (Zheng et al., 2012) with complementary custom R scripts. The R package *StrataG* (Archer et al., 2016) was used to estimate the effective population size to be used in the phasing algorithm. The genotype imputation and phasing were performed using *Beagle* version 5.1 (Browning et al., 2018) using the following parameters: window size of 100 markers, overlap of 20 markers, effective population size of 25, and 1000 interactions.

Additional filters were applied over the imputed SNP data set to finalize the data processing step: SNPs with a minor allele frequency ≤1% across all samples were removed from the downstream analysis. Then, the pairwise linkage disequilibrium (*r* ^2^) was estimated using the R package *SNPRelate* (Zheng et al., 2012). The values of *r* ^2^ were used to generate two different SNP data sets: an LD‐pruned data set containing markers with pairwise *r* ^2^ ≤ 0.5 to be used for population structure analysis and a complete SNP data set to be used for all remaining analyses.

### 2.3 Population structure

We performed DAPC using the LD‐pruned data set, and the linear discriminant analysis (LDA) was initially calculated using the groups defined a priori (Table 1). Then, the procedure was repeated without supplying the LDA with the groups defined a priori. The groups defined for the second LDA were estimated using the *k*‐means clustering algorithm. The DAPC was performed using the R package *adegenet* (Jombart et al., 2010).

### 2.4 Signatures of selection based on site frequency spectrum and genetic differentiation

We calculated the *F*_ST_ (Weir & Cockerham, 1984) between the contrasting populations before and after artificial selection (i.e., by comparing ancestral and elite lines) within each gene pool (AGP and CGP) for each SNP marker using the software *VCFtools* (Danecek et al., 2011). Complementarily, we calculated the site nucleotide diversity (*π*) for the ancestral and elite populations using the software *VCFtools* (Danecek et al., 2011). The *π* values were then used to calculate the *π*‐ratio (ancestral/elite). SNP markers in which the *F*_ST_ values or *π*‐ratio values were greater than the 99th percentile of each metric were considered candidates for positive selection.

The Tajima's *D* statistics (Tajima, 1989) were calculated over 100,000 bp windows with 10,000 bp steps for all populations using the software *VCF‐kit* (Cook & Andersen, 2017). Tajima's *D* values ≤−2 were considered candidates for positive selection. We additionally detected outlier SNPs using *PCAdapt* (Luu et al., 2016). The PCA approach was used to ascertain the differences between populations; then, outliers were detected based on the Mahalanobis distance between the individual *Z*‐scores and the mean.

### 2.5 Signatures of selection based on haplotype extension methods

Three metrics were used to identify signatures of selection based on extended haplotype homozygosity (EHH): cross‐population extended haplotype homozygosity (XP‐EHH), log‐ratio of integrated EHH (Rsb), respectively, described by Sabeti et al. (2007) and Tang et al. (2007), and hapFLK described by Fariello et al. (2013). Empirical distributions of these three estimates were used to estimate the significance of the computed values. XP‐EHH and Rsb calculations were performed with the aid of the *rehh* (Gautier & Vitalis, 2012) package implemented in the R programming language and environment (R Core Team, 2023). The hapFLK estimates were calculated using the *hapFLK* software (Fariello et al., 2013). The candidate regions for selection were then tabulated and visualized.

### 2.6 Annotation of significant candidate regions

Lists of known quantitative trait loci (QTLs), genome‐wide association study (GWAS) loci, and predicted genes were retrieved from SoyBase (Grant et al., 2010) on March 15, 2021. Significant candidate SNPs identified in at least two contrasting populations or detected by two selection tests were considered regions of high importance. We annotated high‐importance SNPs situated within 0.1 cM from the center of the QTL interval, 1 kbp from GWAS loci, and 1 kbp from the predicted genes intervals. The genetic distances were estimated based on the genetic linkage map described in Song et al. (2016) using loess regression.

## 3 RESULTS

### 3.1 Genotyping and data processing

The genotyping using SoySNP50K resulted in a total of 42,289 raw SNPs, of which 42,080 SNPs were mapped to chromosomes, while the remaining 209 SNPs were mapped to unplaced scaffolds. After applying the quality control filters, 35,083 high‐quality SNPs were retained in the final data set.

### 3.2 Population structure

**FIGURE 1:**
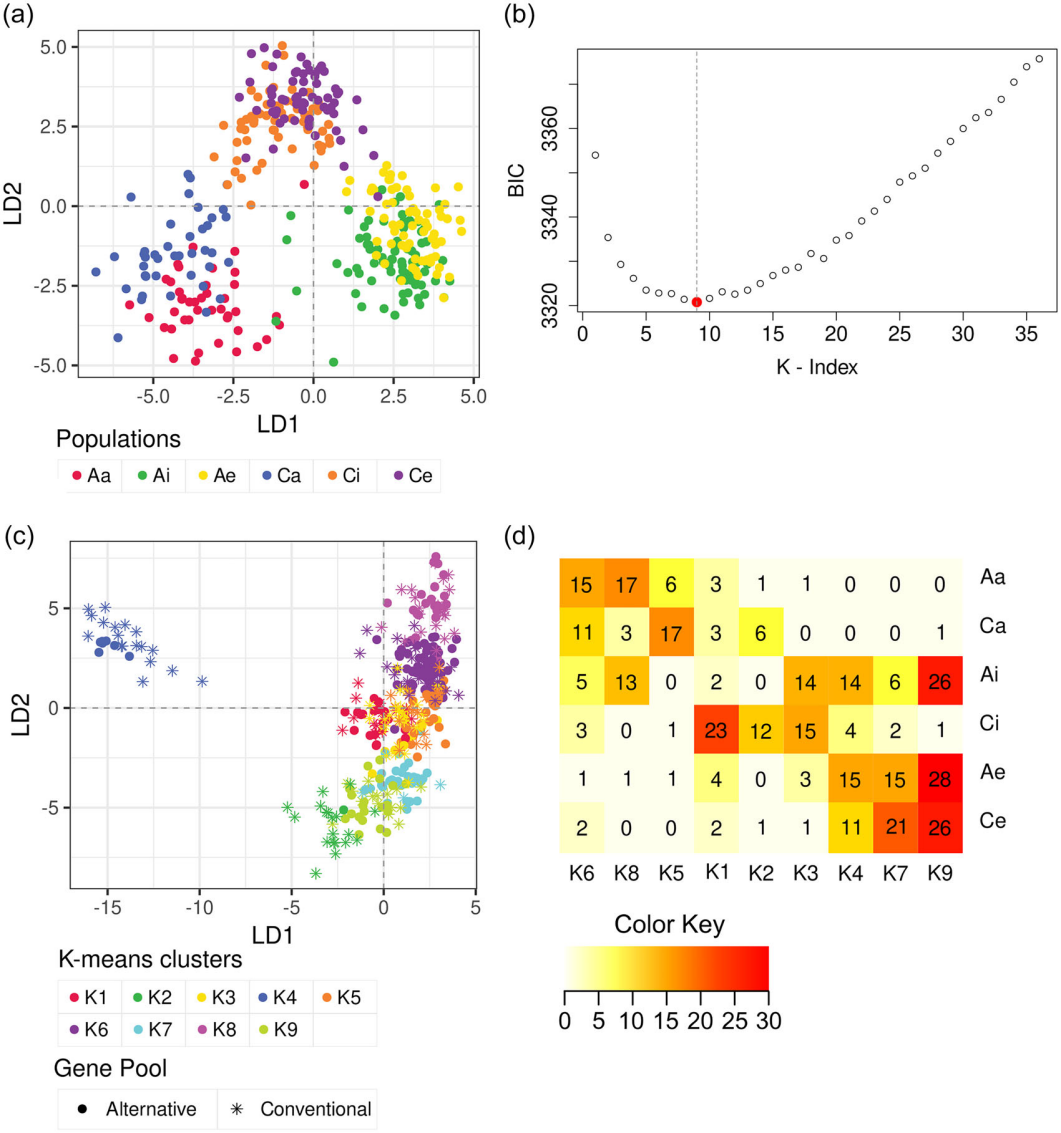
Population stratification based on discriminant analysis of principal components (DAPC). (a) DAPC of soybean germplasm accessions at different stages of breeding selection from the two soybean gene pools, using pre‐defined breeding pools as groups. (b) Number of groups determined by the elbow method for *K*‐means clustering. (c) DAPC based on the groups identified using K‐means clustering. (d) Relation between pre‐defined populations and the groups identified by the *K*‐means clustering algorithm. Aa, alternative gene pool—ancestral population; Ae, alternative gene pool—elite population; Ai, alternative gene pool—intermediate population; Ca, conventional gene pool—ancestral population; Ce, conventional gene pool—elite population; Ci, conventional gene pool—intermediate population.

DAPC revealed the patterns of population structure in the CGP versus AGP (Figure 1). The results clearly show the known relationships among the three stages of selective breeding—ancestral, intermediate, and elite—and the two gene pools—AGP and CGP (Figure 1a). The ancestral pools were slightly intermingled, but the intermediate and elite pools had almost no overlap.

The *K*‐means clustering divides the samples into nine distinct subclusters, as demonstrated by the inflection point in Figure 1b. These subclusters can be further structured in the three stages of selective breeding throughout the second linear discriminant (Figure 1c). As expected, an incomplete stratification between the AGP and CGP was observed within each *K*‐means cluster (Figure 1c,d) due to the common parentage between the two populations. However, based on the predefined breeding pools, the AGP elite population is almost completely separated from the CGP elite population (Figure 1a).

### 3.3 Signatures of selection identified via site frequency spectrum and EHH‐based methods

The number of candidate SNPs under selection estimated using *F*_ST_ values between Ancestral and Elite populations was 339 in the AGP and 358 in the CGP, which was close to the number of candidate SNPs under selection identified using *π*‐ratio: 337 in the AGP and 367 in the CGP. However, the site overlap between the results of these two metrics was very low (Figure S1).

In contrast to the results for F_ST_ and *π*‐ratio, the Tajima's *D* statistic revealed few loci with *D* ≤ −2, and genome‐wide analysis of Tajima's *D* statistic indicates several loci with *D* > 1, or even *D* > 2 across all populations (Figure S2). On the other hand, the PCAdapt algorithm identified 518 significant loci in the AGP and 187 in the CGP.

Several candidate regions under selection were also detected based on the EHH methods. In summary, XP‐EHH identified 374 potential targeted regions in the AGP and 103 in the CGP. Similarly, Rsb detected 345 candidate loci in the AGP and 292 in the CGP. The hapFLK results were more distinct than the other statistics in both gene pools: 236 candidate regions under selection were identified in the AGP and 543 in the CGP.

The tabular results of the signatures of selection detected in the soybean populations addressed in this study are given in Table S2. Multiple significant signatures were detected, including several in common between EHH and SFS methods and between the AGP and CGP selection schemes.

### 3.4 Meta‐analysis for detecting signatures of selection in breeding populations

**FIGURE 2:**
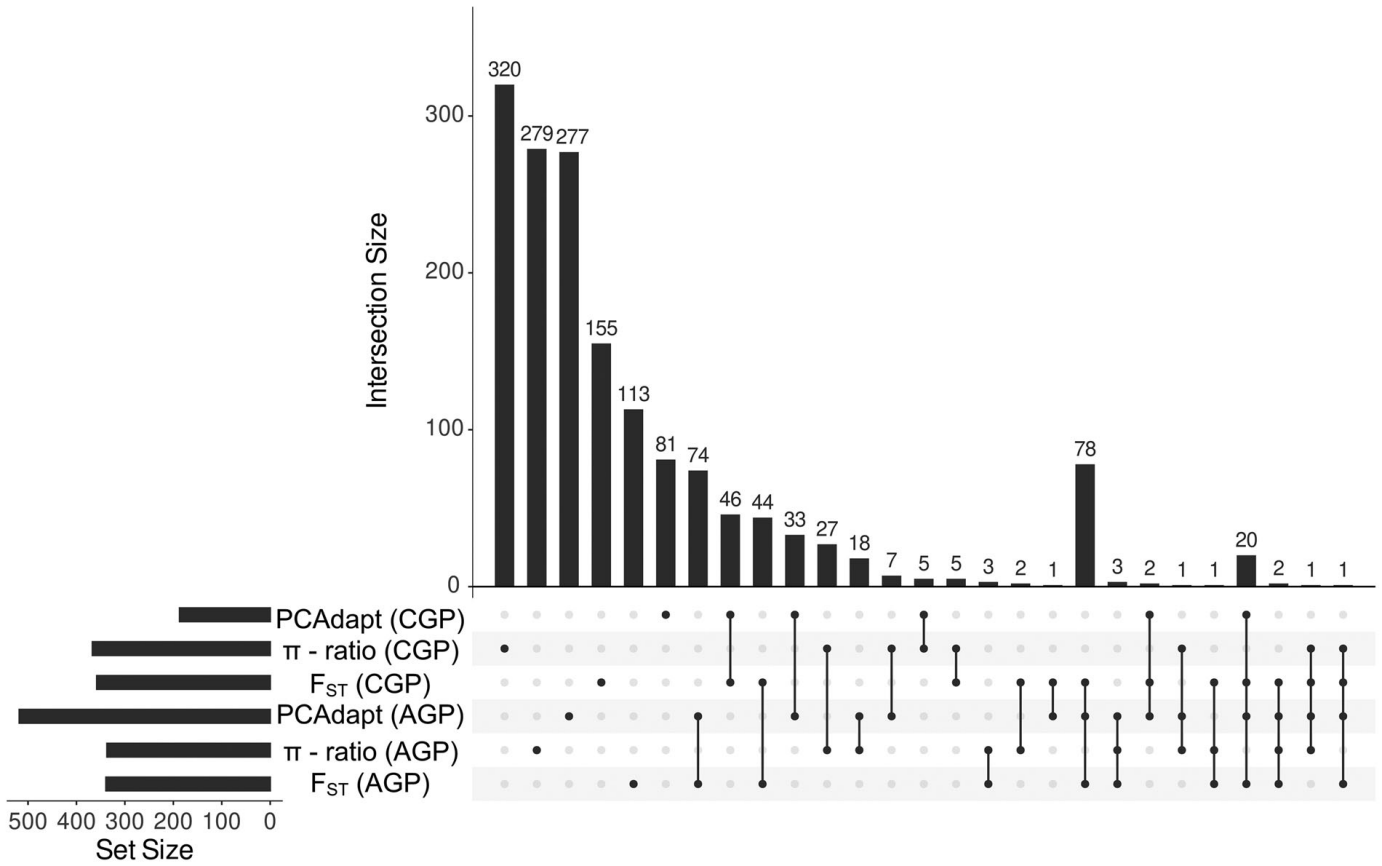
Shared, significant signatures of selection detected by combinations of the three single‐site frequency‐based approaches. CGP, conventional gene pool.

Our analysis yielded a high‐confidence set of putatively selected regions across both gene pools. Figure 2 shows the number of disjoint sets and intersections between the SFS‐based approaches to detect significant signatures of selection in the soybean populations. Most of the disjoint sets of SFS‐based candidate regions were seen in the CGP using the *π*‐ratio approach.

**FIGURE 3:**
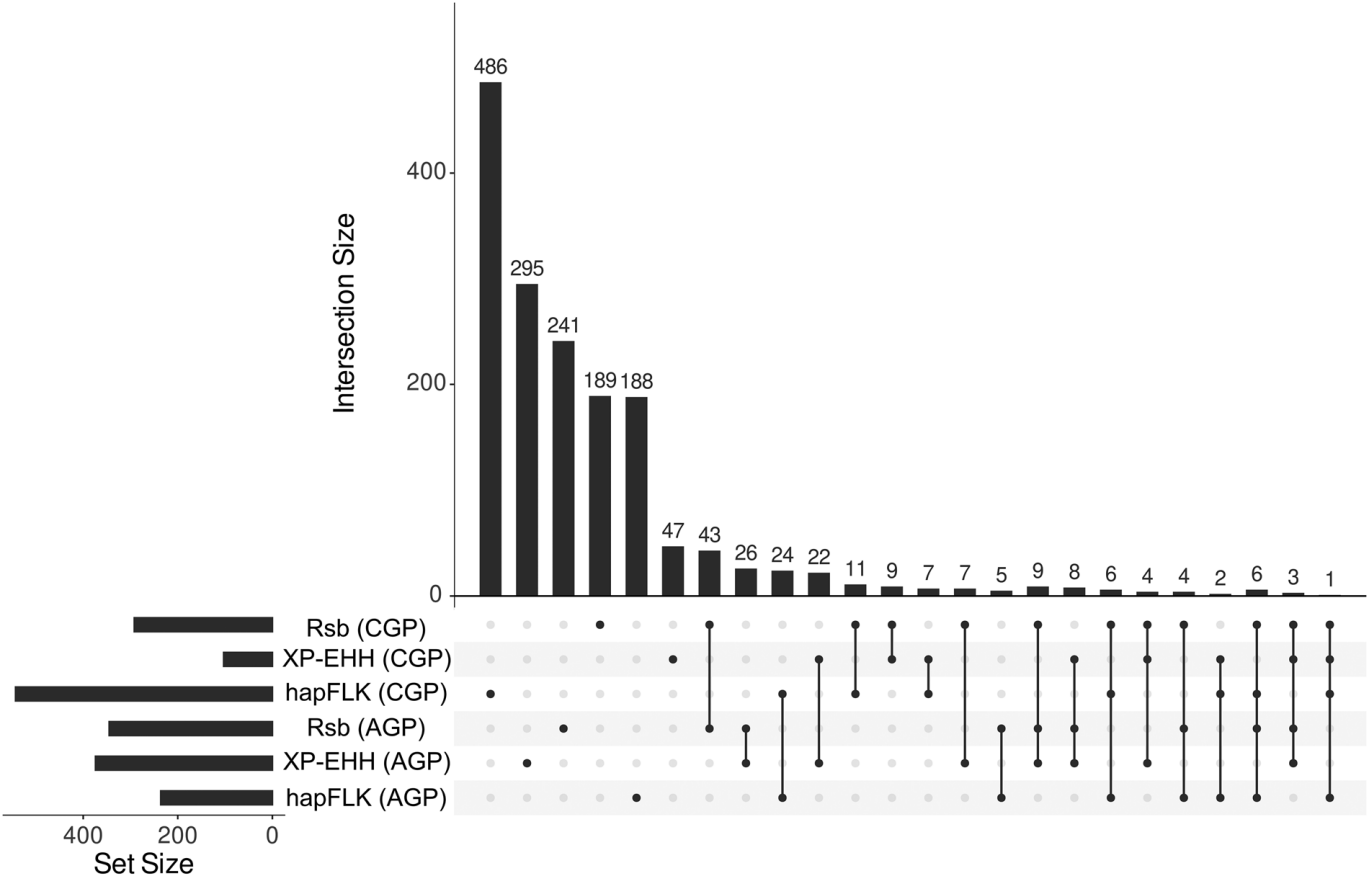
Shared, significant signatures of selection detected by combinations of the three haplotype extension‐based approaches. CGP, conventional gene pool; EHH, extended haplotype homozygosity.

Figure 3 shows the number of disjoint sets and intersections between the EHH‐based methods. The hapFLK method in CGP and XP‐EHH method in AGP yielded most of the disjoint sets observed in the haplotype‐based results. Similar to SFS‐based approaches, no region was identified in both gene pools using all EHH‐based approaches adopted in this study.

Several high‐importance SNPs were identified across gene pools and selection scan tests. The number of candidate regions detected by *F*_ST_ and PCAdapt was more consistent between the gene pools, and a more significant proportion of candidate regions were present in both gene pools relative to the individual pools than for the haplotype extension methods.

### 3.5 Signatures of selection underlying QTL controlling economically important traits

**FIGURE 4:**
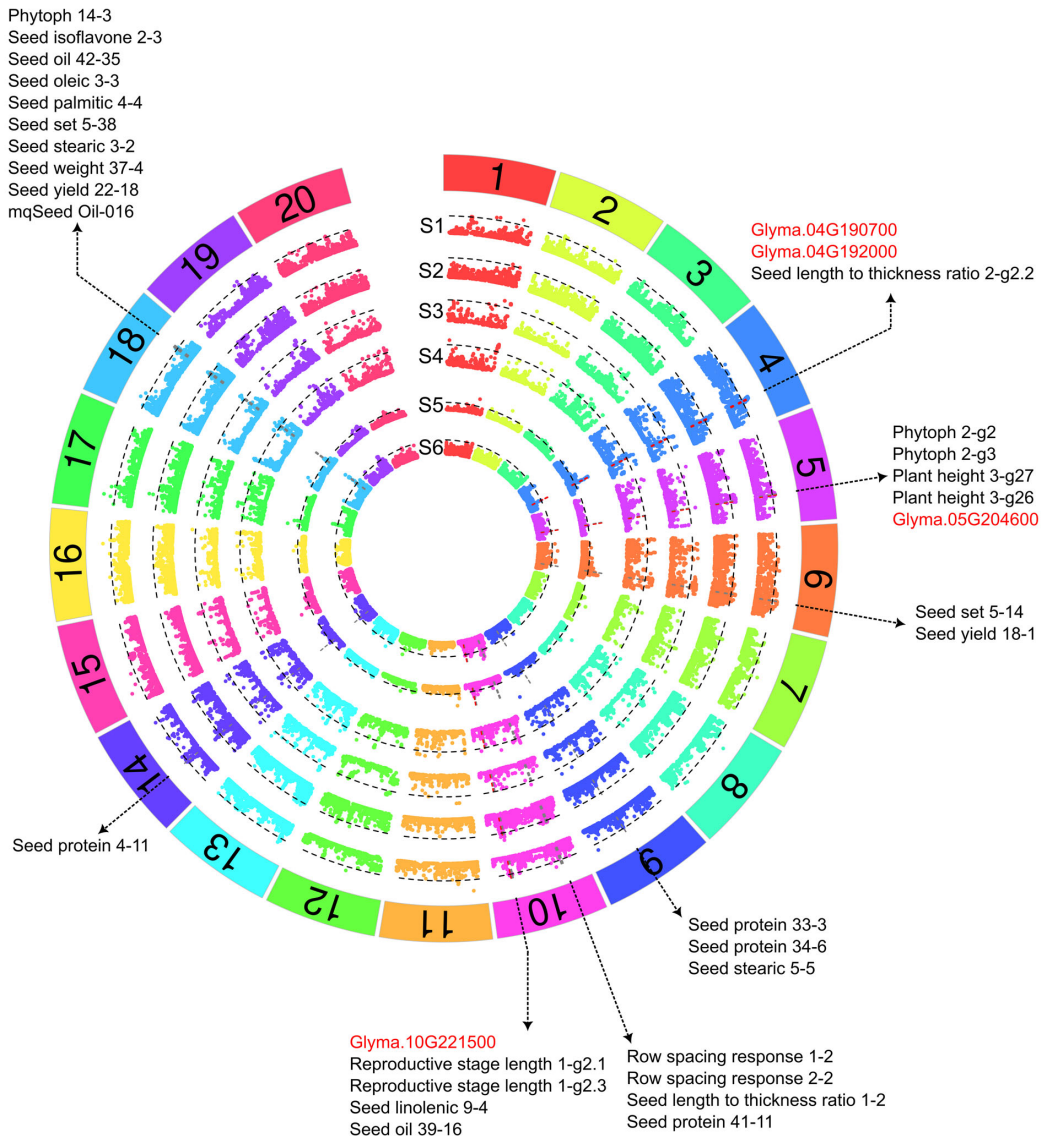
Circular Manhattan plot showing the signatures of selection detected between soybean populations, with selected annotated quantitative trait locus (QTL). Six tracks contain the results from three site frequency spectrum (SSF)‐based selection scan methods performed over both gene pools. S1—*F*_ST_ in alternative gene pool (AGP); S2—*F*_ST_ in conventional gene pool (CGP); S3—*π*‐ratio in AGP; S4—*π*‐ratio in CGP; S5—PCAdapt in AGP; S6—PCAdapt in CGP. Single nucleotide polymorphisms (SNPs) above the thresholds (black dashed lines) were considered significant candidate loci under positive selection (S1 and S2: *F*_ST_ ≥ 99th percentile; S3 and S4: *π*‐ratio ≥ 99th percentile; S5 and S6: *p* ≤ 0.01). Gray vertical lines highlight significant selection peaks located within the same haplotype block as the center of known QTL intervals. Red vertical lines highlight significant selection peaks located within the same haplotype block as selected gene models.

**FIGURE 5:**
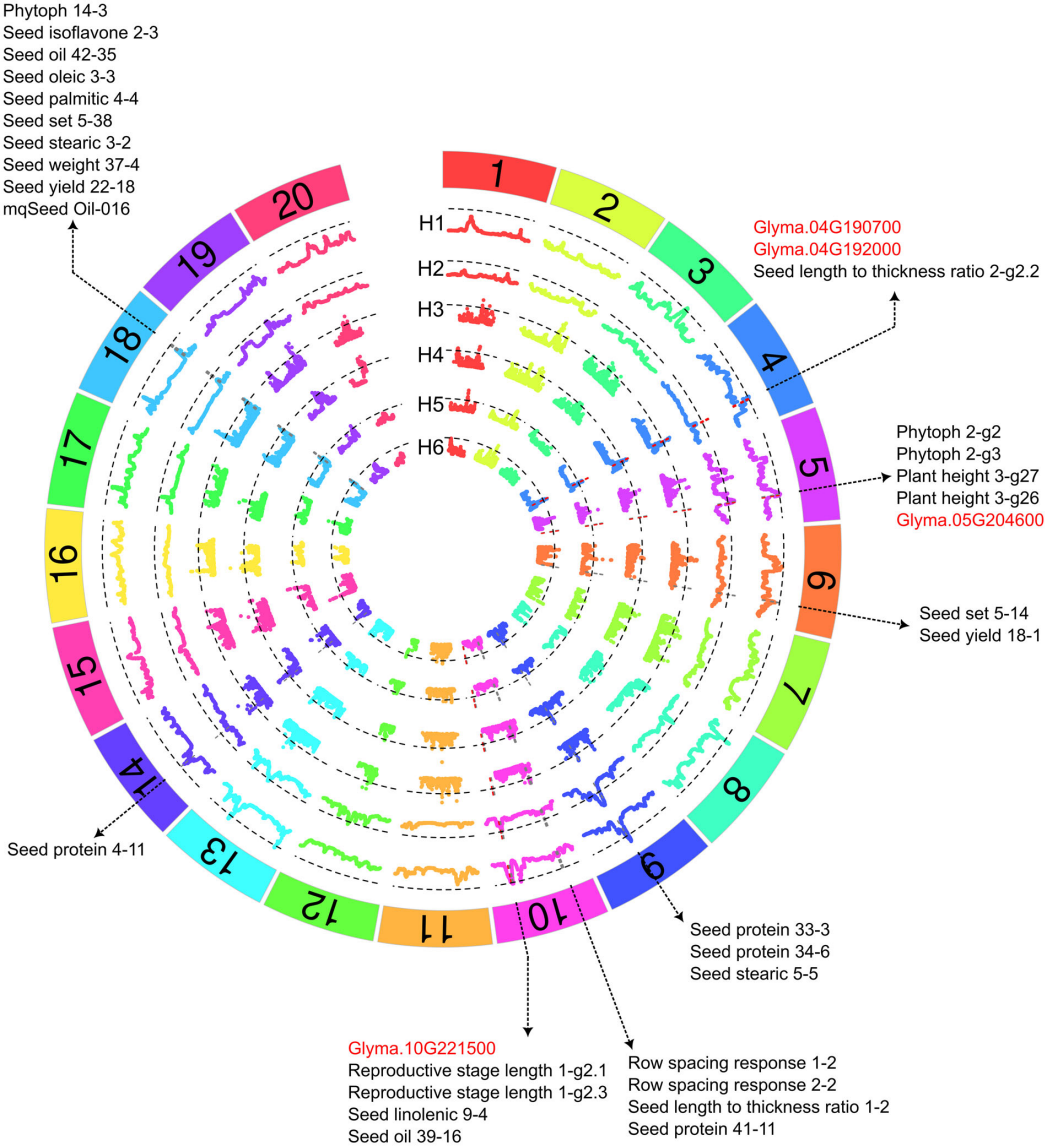
Circular Manhattan plot showing the signatures of selection detected between soybean populations, with selected annotated quantitative trait locus (QTL). Six tracks contain the results from three extended haplotype homozygosity (EHH)‐based approaches performed over both gene pools. H1—hapFLK in alternative gene pool (AGP); H2—hapFLK in conventional gene pool (CGP); H3—Rsb in AGP; H4—Rsb in CGP; H5—XP‐EHH in AGP; H6—XP‐EHH in CGP. Haplotypes above the thresholds (black dashed lines) were considered significant candidate loci under positive selection (*p* ≤ 0.01). Gray vertical lines highlight significant selection peaks located within the same haplotype block as the center of known QTL intervals. Red vertical lines highlight significant selection peaks located within the same haplotype block as selected gene models.

We next compared candidate regions under selection to reported intervals of QTL for economically important soybean traits, using both SFS‐ and EHH‐based methods (Figures 4 and 5). We noticed that for the set of SNPs putatively under selection, several underlie known regions associated with key traits for soybean breeding. We found a total of 538 candidate SNPs under selection that match known QTL. Of these, 141 candidate regions showed significant values only in AGP, while 64 candidate regions showed significant values exclusively in CGP (Table S3). Moreover, we identified 43 candidate loci underlying published GWAS loci (Table S4). A total of 339 high‐importance candidate SNPs flank soybean gene models (Table S5). Table S2 contains numerical details of the results presented in Figures 4 and 5.

**FIGURE 6:**
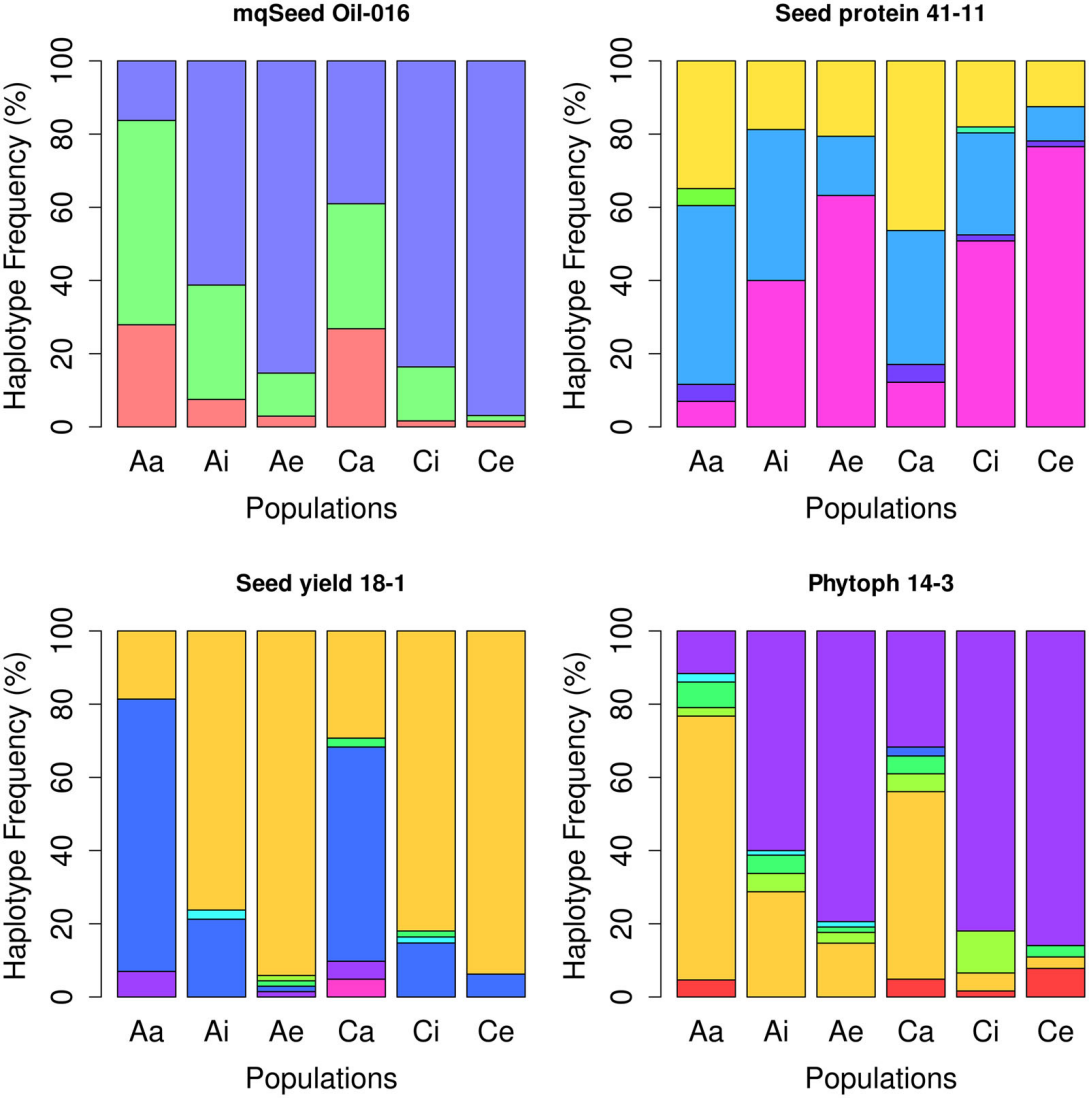
Haplotype frequencies in four haplotype blocks under selection flanking the center of quantitative trait locus (QTL) intervals in title of each sub‐panel, relative to the Wm82.a2.v1 reference genome: (a) haplotype block on chromosome 18 from position 50,590,190 to 50,600,715; (b) chromosome 10 from 4,744,786 to 4,766,269; (c) chromosome 6 from 47,416,685 to 47,448,513; (d) chromosome 18 from 51,667,131 to 51,714,486.

The frequency spectra of the haplotypes under selection that overlapped the center point of known QTLs were then examined for selected QTL. In Figure 6, the relative frequencies of the haplotype blocks flanking the QTL mqSeed Oil‐016, Seed protein 41‐11, Seed yield 18‐1, and Phythoph 14‐3 are shown by population and breeding stage.

## 4 DISCUSSION

In this study, we identified common haplotype‐level frequency changes in two separate soybean gene pools and showed evidence that these changes were caused by the selective breeding process. Soybean is a species with significant socioeconomic impact and a key species for food security, and thus the continued progress of soybean selective breeding is of worldwide importance. The experimental design adopted in this study allowed the identification of common signatures of selection across two genetically distinct soybean gene pools. The populations were independently subjected to selective pressures for economically important traits, such as MG and yield. They are each composed of three subpopulations of soybean lines at different breeding stages, allowing the progression of selection to be followed. Since the two populations were both under selection for increased yield in the North‐Central region of the United States, it is a reasonable assumption that the selective pressures were similar. Therefore, our study provides a distinct perspective for interpreting artificial selection effects on the qualitative and quantitative traits that vary within the two ancestral gene pools. For the 389 loci where we observe selection in the same direction in both gene pools (Table S2), the explanation of genetic drift becomes much less likely, and we conclude that these loci were under artificial selection during the process of soybean breeding for improved yield in the North‐Central United States.

The selective pressure applied to the ancestral populations during the selective breeding of elite soybean lines caused obvious genetic differentiation. Consequently, a clear population structure differentiates the ancestral, intermediate, and elite populations (Viana et al., 2022). This is strong evidence that selection and genetic drift have modified standing genetic variation across several generations of breeding. We also observed that the selection efforts were computationally separable, with the first discriminant function explaining the parallel selection of both populations, while the second discriminant function differentiating the two gene pools. At the risk of oversimplifying the process, the first discriminant function may largely represent common selective pressure, while the second may represent either drift or selection pressures that differ between populations.

The primary hypothesis addressed in this study is that the independent selective breeding programs of the conventional breeding programs and the USDA‐ARS alternative pool caused different, but overlapping, combinations of favorable alleles to increase in frequency, despite being under selection for broadly the same traits and adaptation. Jun et al. (2011) studied the signatures of selection in the soybean genome using microsatellite markers, and they were able to detect signatures of selection in regions adjacent to known QTLs reported in scientific literature associated with yield and disease resistance (albeit without the control of parallel gene pools under selection to eliminate drift). We anticipated that the two initial pools would contain different favorable haplotypes that confer similar, agronomically desirable phenotypes. We also anticipated that some regions, while independently selected, would show enrichment and/or fixation of the same haplotype in both gene pools and that these haplotypes could, with high confidence, be shown to be under positive selection rather than subject to drift. Based on overlap with known QTL, we could then assess whether these haplotypes likely included QTL controlling economically important traits. We conclude that the results support this hypothesis, since we identified a set of candidate regions that were concomitantly under selective pressure in both gene pools, some of which match up with known QTL intervals. The results also corroborate previous studies that reported an association between QTL and several economically important traits, such as seed morphology, seed composition, MG, disease resistance, and plant architecture (Chang & Hartman, 2017; Dhanapal et al., 2018; Fang et al., 2017; Mao et al., 2017).

Santos et al. (2022), studying the genomic signatures of selection in Brazilian and U.S. soybean populations, identified a significant contribution of SNP markers underlying MG loci (*E1*, *E2*, and *FT2a*) to the genetic differentiation (*F*_ST_) between northern and southern germplasm. Correspondingly, our analysis detected signatures of positive selection underlying the *E2* gene *Glyma.10g221500* (Wm82.a2.v1) (Watanabe et al., 2011) in the subpopulations from both gene pools. The soybean MG corresponds to the plant's responsiveness to photoperiod, a critical trait in soybean adaptation. The importance of MG in soybean breeding also lies in its influence on grain yield and seed composition (Ortel et al., 2020; Salmerón et al., 2022). However, our analysis did not detect significant signatures of selection underlying other *E* series genes known to date, possibly because the major ancestral lines have similar maturity to the elite cultivars.

Notwithstanding, our study revealed signatures of selection in regions controlling other important developmental genes, such as growth regulating factors (*GRF; Glyma.01g234400*), stem termination (*Dt1*; *Glyma.19g194300*), and acetolactate synthase genes *(ALS; Glyma.04g196100*). *GRF* is an important class of plant‐specific transcription factors involved in multiple developmental processes, including the balance between stress response and plant growth (Omidbakhshfard et al., 2015). Moreover, variations in the gene *Dt1* determine the growth habit in soybeans (Liu et al., 2010) and have a strong influence on lodging (Hwang & Lee, 2019), a trait target during the soybean improvement in AGP and CGP. Similarly, specific combinations of *ALS* alleles can confer plant tolerance to herbicides (Walter et al., 2014), and targeting novel *ALS* alleles that confer herbicide resistance can benefit modern agricultural applications (Kuang et al., 2020). Therefore, our results indicate that throughout different eras of soybean genetic improvement, the breeding efforts also contributed to selecting beneficial alleles for traits that can play a part in soybean's adaptation to breeding environments.

High genomic marker coverage offers an opportunity to increase detection power by identifying regions under selection by extended haplotypes through correlations between adjacent markers (Fariello et al., 2013). This procedure provides an approach to identifying haplotypes, rather than individual polymorphisms, that are under selection, and identifies fewer false positive regions than allele frequency metrics that aim to measure fixation or disequilibrium. The signal is detectable due to the selective sweep effect, where changes in allele frequency and linkage disequilibrium extend to neighboring regions around the selected locus (Jun et al., 2011; McVean, 2007). Our coverage using the 50K Illumina array was sufficient to identify haplotypes under selection containing multiple marker loci.

Using the EHH‐based metrics, we identified many haplotypes showing signatures of selection in both gene pools. The process of directional selection contributes to the fixation of specific haplotypes in elite populations. These results corroborate the findings of Zhao et al. (2015) on allele fixation due to soybean breeding. Zhao et al. (2015) reported that the fixation of alleles in elite soybean lines had been accelerated by artificial selection associated with the bottleneck events that occurred during the species' domestication. However, drift also plays a critical role in changes in haplotype frequency in finite populations (Vaughn & Li, 2016). In the current study, the combination of the results from independent allele‐ and haplotype‐based selection metrics, the association of the candidate haplotypes with previously reported QTL, and most importantly, the analysis of loci under parallel and concomitant selection in both gene pools, allowed the number of false positives to be minimized.

As one example, a strong signature of selection on chromosome 4 was observed in several SFS‐ and EHH‐based selection scan approaches; thus, we conclude this locus is under positive selection in both gene pools. The fact that this region was detected regardless of the method, and gene pool aroused our interest in the changes in haplotype diversity underlying this locus. The region flanks a QTL controlling a trait responsible for seed morphology (Fang et al., 2017). Seed morphology traits can be essential components of yield (Tao et al., 2017), and knowledge of the genetic architecture of these traits offers an opportunity to increase the efficiency of genetic improvement of yield‐related traits. In both gene pools, there was a strong selective sweep in this region, and the fact that the same haplotype was strongly selected by two independent breeding programs indicates this QTL is a crucial component of economic traits under selection in the Midwest. This represents one of the several examples of parallel selection in the two gene pools detected by the selection scan approaches.

Moreover, we identified signatures of selection adjacent to regions associated with several disease‐resistance traits. Significant signatures of positive selection underlying QTLs and GWAS loci associated with resistance to the fungus *Phytophthora sojae* were identified in both gene pools. As one example, Qin et al. (2017) reported a 33‐kbp genomic region harboring candidate haplotypes detected in our study (Table S4) that is significantly associated with resistance to *Phytophthora* race 1. The resistance to *Phytophthora sojae* is a critical trait to prevent yield loss in regions affected by this pathogen since this species threatens soybean fields in any step of soybean production (Dorrance, 2018).

The main goal of most soybean breeding programs is to develop high‐yield soybean lines, and this goal is usually achieved by selecting the best‐performing individuals from the breeding germplasm pool as parents for the next generation. Although the elite populations used in our study were all under selection for high yield, we demonstrated several putative regions under selection that do not show significant signatures of selection in both gene pools. Although drift is a possible explanation for this, some of these candidate regions correlate with yield‐related QTLs reported in the literature (Du et al., 2009; Fang et al., 2017; Palomeque et al., 2010). Thus, some differential selection due to the different starting gene pools and different environments of the breeding programs for the separate pools is also likely.

In most of our analysis, we found several loci under selection to be in common between gene pools, and that those not meeting a shared significance threshold still often proceeded to fixation in the same way (Figure 6). This is despite the fact that the AGP was specifically designed to maximize new parentage and diversity in the starting materials. This finding validates our approach and indicates a range of loci that could be selected more rapidly, perhaps using genomic selection techniques. However, this also represents another warning that the diversity of new, agronomically useful alleles is limited in the cultivated soybean population.

These results are evidence of selection in yield‐related haplotypes and show that these haplotypes can be identified through selection analysis, which could help increase the efficiency of breeding programs. Usually, the implementation of marker‐assisted selection (Qian et al., 2017) or genomic breeding (Lin et al., 2020) relies on genotype–phenotype association in specific environments to identify desirable haplotypes. We suggest that adding selection analysis to the toolkit used to determine loci that should be combined in elite lines may increase the efficiency of these methods.

In addition to the selection of known QTL, our results support the hypothesis that in different gene pools, different haplotypes have been fixed by selection for the same trait. The promising results of these analyses may help in the selection of contrasting parentage in the further development of elite lines, for example, by combining multiple haplotypes conferring advantageous characteristics. Direct identification of all the haplotype blocks corresponding to QTL responsible for key traits should eventually be possible using the comparative selection methods employed here, and as such this effort has direct relevance for the improvement of the yield and resilience in modern elite cultivars (Qian et al., 2017). The results presented in our study can therefore support the development of new higher yielding soybean lines via a new way to identify desirable haplotypes for genomic selection. Our results also offer a caution that many, if not most, of the loci proceeding toward selection are common between these gene pools, despite the AGP being purposely selected for diverse ancestry. This raises concerns that the potential for significant further yield increases via selection on the current soybean population may be limited.

## AUTHOR CONTRIBUTIONS

**João Paulo Gomes Viana**: Conceptualization; formal analysis; investigation; methodology; validation; visualization; writing‐original draft; writing‐review and editing. **Arián Avalos**: Methodology; formal analysis. **Zhihai Zhang**: Formal analysis. **Randall Nelson**: Conceptualization; resources; writing—original draft. **Matthew E. Hudson**: Conceptualization; formal analysis; methodology; project administration; supervision; writing—original draft; writing—review and editing.

## CONFLICT OF INTEREST STATEMENT

The authors declare no conflicts of interest.

## Supporting information

Supplementary Table 1 – List of populations and accessions from alternative gene pool (AGP) and conventional gene pool (CGP)

Supplementary Table 2 – Genome‐wide scans for signatures of selection in contrasting populations (Ancestral, Intermediate, and Elite) of independent soybean gene pools (Ancestral vs. Conventional).

Supplementary Table 3 – List of QTLs underlying candidate SNPs under positive selection.

Supplementary Table 4 – List of GWAS loci underlying candidate SNPs under positive selection.

Supplementary Table 5 – List of gene models flanked by candidate regions under selection.

Supporting Information

Supporting Information

## Data Availability

The data used in this study was previously made public as supplemental material in the following publication: https://doi.org/10.1007/s00122‐022‐04056‐5
